# Neuroprotective potential of *Mentha piperita* extract prevents motor dysfunctions in mouse model of Parkinson’s disease through anti-oxidant capacities

**DOI:** 10.1371/journal.pone.0302102

**Published:** 2024-04-16

**Authors:** Rabia Anjum, Chand Raza, Mehwish Faheem, Arif Ullah, Maham Chaudhry

**Affiliations:** Laboratory of Neurobehavioral Biology, Department of Zoology, Government College University Lahore, Punjab, Pakistan; University College London, UNITED KINGDOM

## Abstract

Parkinson’s disease (PD) is the second most common neurodegenerative disease in the world. Neurodegeneration of the substantia nigra (SN) and diminished release of dopamine are prominent causes of this progressive disease. The current study aims to evaluate the protective potential of ethanolic extract of *Mentha piperita* (EthMP) against rotenone-mediated PD features, dopaminergic neuronal degeneration, oxidative stress and neuronal survival in a mouse model. Swiss albino male mice were assigned to five groups: control (2.5% DMSO vehicle), PD (rotenone 2.5 mg/kg), EthMP and rotenone (200mg/kg and 2.5mg/kg, respectively), EthMP (200 mg/kg), and Sinemet, reference treatment containing levodopa and carbidopa (20 mg/kg and rotenone 2.5mg/kg). Behavioral tests for motor functional deficit analysis were performed. Anti-oxidant capacity was estimated using standard antioxidant markers. Histopathology of the mid-brain for neurodegeneration estimation was performed. HPLC based dopamine level analysis and modulation of gene expression using quantitative real-time polymerase chain reaction was performed for the selected genes. EthMP administration significantly prevented the rotenone-mediated motor dysfunctions compared to PD group as assessed through open field, beam walk, pole climb down, stepping, tail suspension, and stride length tests. EthMP administration modulated the lipid peroxidation (LPO), reduced glutathione (GSH), and superoxide dismutase (SOD) levels, as well as glutathione-s-transferase (GST) and catalase (CAT) activities in mouse brain. EthMP extract prevented neurodegeneration in the SN of mice and partially maintained dopamine levels. The expression of genes related to dopamine, anti-oxidant potential and synapses were modulated in *M*. *piperita* (MP) extract treated mice brains. Current data suggest therapeutic capacities of MP extract and neuroprotective capacities, possibly through antioxidant capacities. Therefore, it may have potential clinical applications for PD management.

## Introduction

Parkinson’s disease (PD) is a progressive neurodegenerative disease which affects 0.5–1% people of 65–69 years and 1–3% of above 80 years old [1, 2]. PD is a multifactorial disease with progressive loss of the dopaminergic neurons in the basal ganglia of midbrain, prior to symptoms onset. The primary motor symptoms include slowness of movements, muscular rigidity and resting tremors [3]. PD diagnosis requires pathological confirmation of distinct neuronal inclusions, Lewy bodies (LBs), and a reduction in the number of dopamine (DA) producing neurons in the substantia nigra pars compacta. On average, sporadic PD features onset at the age of 60 years, however rare forms of a familial PD may experience features under the age of 40 years. The estimation of prevalence rate reveals a rapid rise from 1/200 person in the 7^th^ decade to 1/40 person in the 8^th^ decade of life [4].

PD progression is linked with the gradual degeneration of the Substantia Nigra pars compacta (SNc) neurons leading to diminished supplies of DA. This lowered neurotransmission of DA leads to compromised motor functions including bradykinesia, rigidity and often resting tremors [3]. The quality of life in the advance stage of PD worsens, causing huge socioeconomic impact. Pharmacological treatments include gold standard administration of levodopa to compensate the DA levels resulting in symptomatic relief [3]. DA is impermeable to blood brain barrier in contrast to levodopa (a metabolic precursor of DA). Sinemet (Sin) (combination of levodopa and carbidopa) is one of widely used pharmacological drugs to increase the DA availability to CNS owing to inhibition of levodopa to DA conversion in blood circulation.

Rotenone is an insecticide and a popular PD mimetic owing to its blood brain barrier permeability. It selectively impairs the cytochrome I complex functions in SNc DA neurons leading to enhanced reactive oxygen species (ROS) generation and hence neurodegeneration. Rotenone reproduce the parkinsonian symptoms in rodent models, particularly motor deficits [5].

*Mentha piperita* commonly called as peppermint, is a hybrid of *M*. *aquatica* (watermint) and *M*. *spicata* (spearmint). It is a native genus of the Mediterranean region and cultivated all over the world for the use in the flavors, fragrance, medicinal ingredients and pharmaceutical applications. In folk medicine, decoction of MP is used to treat nausea, bronchitis, and anorexia, as an antiseptics, and antifungals and to impart neuroprotection [6, 7]. MP and *M*. *aquatica* impart neuroprotection to PC12 cells against oxidative stress [8]. Antimicrobial capacities of MP extracts revealed significant inhibition of bacterial strains and Candida spp. [9]. Furthermore, essential oils from MP impart anti-nociceptive activity in mice and reduce plasma triglyceride levels in rats experiencing lead-induced toxicity [10]. Modern analytical tools reveal an array of biologically active ingredients in MP extracts [11]. However neuroprotective capacities of MP extract and its ingredients are yet to be determined *in vivo*.

The current study is focused on the ability of MP to protect against rotenone-induced behavioral deficits, dopaminergic degeneration, and oxidative stress and neuronal survival activity in mouse model of PD.

## Materials and methods

### Plant collection and extract preparation

*M*. *piperita* was collected from the District Lahore, Pakistan. The plant was identified (Voucher No. GC. Herb. Bot. 1919) from taxonomic experts at Department of Botany, Government College University Lahore, Pakistan. The leaves were washed, dried and ground into powder. Briefly, 10 grams of dried leaf powder was suspended in 100 mL of ethanol (Sigma-Aldrich, Cat. # 32221) (1:10 ratio). The solution was kept on the orbital shaker at 200 rpm for 24 hours. Filtration was performed using Whatman filter paper. The filtrate was evaporated at 40°C and finally lyophilized and stored at 4°C until further use.

### Gas Chromatography−Mass Spectrometry (GC-MS) analysis of ethanolic extracts of *M*. *piperita*

GC-MS is the most common technique used in the laboratory harnessing mass spectrometry to identify the compounds present in the sample. GC-MS usually separates the volatile compunds from the sample and MS identifies the compunds on the basis of their molecular masses. GC-MS was run by vapourizing the analyte while maintaining the temperature between 30 to 300°C. Briefly, the analysis was made using GC 7890A (Agilent, USA) with mass spectrometer (5975C) coupled to a split-splitless injector and a HP-5MS fused silica capillary column (5%phenyl 95% dimethylpolysiloxane, 30m × 0.25mm i.d., film thickness of 0.25 μm). Column was operated at 250°C injetor temperature. Isotherm was hel at 50°C for 2 minutes, followed by a ramp of 7°C/min to 290°C, further isotherm held for 1 minute, a second ramp to 280°C at 20°C/minute and finally an isotherm held for 37 minutes. 1μL extract was injected (split flow ratio 5:1), helium carrier gas (constant rate 1ml/minute) for the analysis. Electronic-impact ion source and MS quadrupole temperatures were adjusted at 230°C and 150°C and mass spectra were acquired (EI mode with scan range of m/z 35–500 and ionization energy of 70eV). Then, the chromatogram was evelauted on the basis of retention time. GC-MS also showed the mass of given particles to their electrostatic charges. It uses the principle of chemical ionization and electron impact techniques. The GC-MS uses parameters such as retention time, initial time, final time, area, percentage area, height and percentage height (Chauhan and Chauhan, 2014).

The identification of bioactive components of EthMP was performed by matching their mass spectra with the library entries of mass spectra databases (NIST-08 MS library version 20).

### Animal housing and grouping

Adult male Swiss albino mice (8-weeks old, 24–28 grams in weight) were purchased from the animal housing facility of Government College University, Lahore, Pakistan. Mice were housed in standard cages at 25±2°C, 12h light and dark cycles with unlimited supplies of rodent diet and water. The study was conducted following approval (Ref. # GCU-IIB-01/2023) from Institutional Bioethics Committee of Government College University, Lahore. All the experiments were performed in accordance with the ARRIVE (Animal Research: Reporting of In Vivo Experiments) guidelines.

Mice were subcutaneously (s.c) injected with 2.5 mg/kg of rotenone (Oakwood Chemicals. CAS # 83-79-4) daily for 21-consecutive days to induce the PD symptoms as stated in the previous researches [12–14]. EthMP extract (200 mg/kg) was administered orally, 1 hour prior to rotenone administration for 21 consecutive days with selected dose from the previous work [13, 15–17]. For positive control group reference drug, Sinemet (20mg/kg) was administered orally for 21-consecutive days with slight modifications from previous studies [18–20], 1 hour prior to rotenone administration. The mice were acclimatized for one week and trained for the neurobehavioral assessments. A baseline reading was conducted before the dosing and extent of PD symptoms were assessed following 21-days of rotenone administration. Mice were randomly assigned into five groups as following,

Group 1: Control, 2.5% DMSO (s.c)
Group 2: Rotenone group (2.5mg/kg, s.c)
Group 3: EthMP (200mg/kg, oral) + Rotenone (2.5mg/kg, s.c) group
Group 4: EthMP group (200mg/kg, oral)
Group 5: Sinemet (20mg/kg, oral) + Rotenone (2.5mg/kg, s.c) group

### Behavioral test for motor dysfunction analysis

The mice were subjected to six different tests to evaluate the PD symptoms. All the tests were performed blinded to the mice grouping. Mice were habituated and trained for the tests prior to dosing and baseline readings were noted. Following dosing, behavioral tests were performed after 21-days of treatment.

#### Open field test

Open field test estimates the exploratory activities of model animals and is used to measure the effectiveness of a drug on locomotive dysfunction [21]. The experiment was carried out in the wooden apparatus of 50cm x 50cm area with boundary wall height of 50cm. A white sheet with 16 equal squares was floored. The mouse was marked on the neck (with black spot) and placed in the center of box and mice activities were recorded for a period of 5 minutes. The total distance travelled in 5 minutes was measured by the software ToxTrac as described previously [22] and number of entries in squares, marked on the floor were counted manually. The apparatus was cleaned with 70% ethanol before and after the mouse exposure.

#### Beam walk test

The beam walk test is widely used to assess the extent of motor coordination and balance in rodent models of locomotive dysfunctions [23]. It was done using the wooden narrow beam of 1m length and 10 mm diameter, held at the height of 60 cm, parallel to the bench top. The mouse was gently placed on one side of the rod and allowed to travel and time to traverse 1 meter distance was recorded. Each mouse was allowed to walk twice and an average reading was noted for analysis. The cut-off value of 120 seconds was given to those mice that fell or did not travel at all.

#### Pole climb-down test

This test was conducted to access the bradykinesia and the motor coordination [24]. It consists of a vertical wooden rod with rough surface which is 50 cm in height and 1 cm in diameter, fixed on the platform. The mouse was placed on the top of the pole with head facing upwards. The time to turn-down and climb-down to the base were videotaped. The default value of 120 seconds was given to the mice that fail to climb down or slipped down.

#### Stride length

The stride length or gait analysis was used to evaluate the normal gait functions and synchrony of walking [25]. The apparatus was consisting of straight corridor of 2.5 feet long and 8.5 cm wide with boundary walls height of 8cm. The floor was lined with white paper. The hind limbs were painted with water soluble, non-toxin ink and allowed the animal to move. The footprints were air-dried and the distance between the inner toes of the two consecutive hind limbs of same side after a stride was determined using the Vernier caliper.

#### Stepping test

The stepping test is used to access forelimb akinesia in animal [26]. Briefly each mouse was placed on the corridor and allowed to cover a distance of 1 meter. The time taken to cover the distance of 1 meter length was noted.

#### Tail suspension test

It assesses the depression-like behavior in the mouse [26]. The apparatus was made of wood which is 55 cm in height, 60 cm in width and 15 cm deep. The apparatus was divided into four chambers. An aluminum rod was placed on the top on which the mouse was suspended with the help of a tape, and mice were placed in the middle of rod 20 to 25 cm above the floor of apparatus [27]. The suspended 4 mice were videotaped simultaneously, for the period of 6 minutes and were analyzed later to find the immobility time.

### Mice brain procurement

Mice were anesthetized with the combination of ketamine (100 mg/kg) and xylazine (10 mg/kg). Then, the mice were terminally euthanized by the cervical dislocation. Each mouse was decapitated and brain was procured using autoclaved surgical tools in a sanitized area under the dissecting microscope. The mice brain were quickly washed with phosphate buffered saline (ice-cold). One cerebral hemisphere was stored in 10% formalin (for histopathology) while the other hemisphere was frozen with the help of liquid nitrogen for antioxidant tests.

### Antioxidant tests

The standard antioxidant tests were conducted to evaluate the antioxidant capacities of mice brain. The mice brains were powdered with the help of mortar and pestle in liquid nitrogen and suspended in 10% phosphate buffer saline (pH = 7.4). 1ml of homogenate was saved for lipid peroxidation (LPO) while remaining was centrifuged at 13000 rpm for 30 minutes at 4°C to obtain post mitochondrial supernatant (PMS). PMS was stored at -20°C.

#### Lipid peroxidation (LPO)

The estimation of LPO was calculated using a mixture of 10% tissue homogenate, 10% trichloroacetic acid and 0.67% 2-thiobarbituric acid in equal volumes. The mixture was heated (45 minutes at 100°C) and the supernatant was isolated at 6000g centrifugation [28]. The supernatant was processed for absorbance measurements at 532nm. The colorimetric differences owing to thiobarbituric acid (TBA) components were analyzed for estimation of LPO levels. The extinction coefficient of 1.56x 105/M/cm was used for calculation of LPO level presented as μmol/g of tissue.

#### Reduced Glutathione (GSH)

200μL of PMS was mixed with sufosalicyclic acid (4%) and incubated for an hour (at 4°C). 0.1M phosphate buffer (pH 7.4) and 10nM DTNB (5,5’-dithiobis-2-nitrobenzoic acid) were added to the separated supernatant (3000g). Absorbance was recorded at 412nm and results were presented as nmol GSH/g of tissue.

#### Glutathione-S-Transferase (GST)

The GST activity was estimated using CDNB (1-chloro-2,4-dinitrobenzene) substrate as reported previously [29]. PMS (200μl) was mixed with GSH (1mM) and CDNB (1mM) and a total volume of 2ml was exposed to 340nm for absorbance. The GST activity of mouse brain was reported as nmol/min/mg protein.

#### Catalase (CAT)

Hydrogen peroxide (0.09M) was mixed with PMS and phosphate buffer to estimate CAT activity. The absorbance at 240nm at an interval of 1 minute was observed for 3 minutes. The results were presented as nmol/min/mg of protein.

#### Superoxide Dismutase (SOD)

SOD activity was determined by the enzyme source’s ability to block nitroblue tetrazolium. The reaction mixture consists of 0.6% triton X, 60 M nitroblue tetrazolium, and 50 mM sodium carbonate buffer. The addition of 20 mM hydroxylamine hydrochloride started the process. After adding PMS, the nitroblue tetrazolium was seen to be inhibited at 540 nm.

#### Protein estimation

Quantification of proteins was done using the method described previously [30].

### Histopathology of Substantia nigra

The brain of mice was harvested and fixed with 10% formalin. The midbrain region was isolated under dissecting microscope as per mouse brain atlas (1.5mm to 4.5mm from Bregma) as described previously with some modifications [31]. The tissues were impregnated with molten paraffin and blocks were prepared. Slices of 5μm sections were cut with the help of microtome and stained with Hematoxylin and Eosin as previously described [32]. The slides were mounted and imaging was done with camera fitted microscope (ProgRec CT3). The area of multipolar cells was evaluated using ImageJ (Version 1.52a, NIH, USA). Three mice per group, and three sections per mouse were analyzed in ImageJ. Briefly, the images were converted to 8-bit grayscale. Signal to noice ratio was optimized using Image > Adjust > Threshold tools of the software. The images were subjected to nuclear area analysis by navigating to Analyze > Tool > ROI Manager tools. The measurements for area, mean, and circulation were acquired. The percentage neurodegeneration was calculated using morphological features of the cells as described previously [33].

### HPLC of dopamine level in Brain

The dopamine level estimation from mice (n = 3mice/group) midbrain region was performed using HPLC. The midbrain was homogenized in phosphate buffer, centrifuged (10,000g at 4°C) to obtain the supernatant. The dopamine (Merck, H8502) concentration of 0.1 mg/ml was taken as standard. The mixture of mobile phase was 0.01% EDTA (adjusted to pH 4.0, 100% acetic acid), 2% methanol (v/v), and 7% acetonitrile (v/v). All solutions for HPLC analysis were double filtered through 0.2 μm membranes and degassed before use as described previously [34]. The flow rate of mobile phase was 0.8 ml per minute and UV detector was set at 340 nm. The final dopamine level was represented as μg/mg of brain tissue [34].

### Isolation of RNA and cDNA synthesis

The brain procurement was followed with a quick wash in sterilized ice-cold phosphate buffered saline. Brain was homogenized in TRIzol reagent (Sigma-Aldrich, USA) by repeated pipetting of the tissue manually with the help of micropipette tips of decreasing diameter. Isopropanol-chloroform method was used to extract total RNA [35]. Nanodrop based quantification and RNA quality check was performed. 1μg of RNA with 260/280 ratio of 1.8–2 was used for complementary DNA (cDNA) synthesis. cDNA was synthesized using commercially available RevertAid cDNA synthesis kit (Thermo Fisher Scientific, USA). cDNA was then 4 times diluted in RNase/DNase free water and stored at– 20°C.

### Quantitative real-time polymerase chain reaction

Primers used for quantification of Th (Tyrosine hydroxylase), Nrf-2 (nuclear factor erythroid 2–related factor 2), GPx4 (Glutathione peroxidase 4), SOD-2 (Super oxide dismutase 2), DRD-2 (dopamine receptor D2), PGC 1α (Pparg coactivator 1 alpha), synaptophysin and housekeeping gene GAPDH (Glyceraldehyde 3-phosphate dehydrogenase) were synthesized using primer 3 plus (Table 1). CFX 96 (Bio-Rad, Hercules, CA, USA) plates were used to perform real-time qRT-PCR with SYBRgreen probe.

**Table 1:** Primer sequences of selected genes.

Genes		Primer sequence 5′ to 3′
Glyceraldehyde-3-phosphate (GAPDH)	Forward Reverse	CATGGCCTTCCGTGTTCCTA CCTGCTTCACCACCTTCTTGAT
Glutathione peroxidase 4 (GPx4)	Forward Reverse	CCTTCCCCTGCAACCAGTTT CCACGCAGCCGTTCTTATCA
Tyrosine Hydroxylase (Th)	Forward Reverse	GGAGACAGAACTCGGGACCA CGTCCAATGAACCTTGGGGA
Dopamine receptor D2 (Drd2)	Forward Reverse	ACTCAAGGGCAACTGTACCC GGATGGATCGGGGAGAGTGA
Synaptophysin (Syp)	Forward Reverse	AAGGCCTGTCCGATGTGAAG GCCTGTCTCCTTGAACACGA
Pparg coactivator 1 alpha (Pgc-1α)	Forward Reverse	GTGCTGTGTGTCAGAGTGGA TTCCGATTGGTCGCTACACC
Superoxide dismutase 2 (Sod2)	Forward Reverse	TCTTTGGCTCATTGGGTCCT CAGATAAACAGGGGCTTCGC
Nuclear Factor-erythroid 2 Like 2 (Nrf2)	Forward Reverse	ACATGGAGCAAGTTTGGCAG TGGAGAGGATGCTGCTGAAA

#### Data analysis

The mRNA expression was calculated as fold change relative to control using formula 2^−ΔΔCt^ [36] where ΔCt = Ct target gene- Ct reference gene. Software generated cycle threshold values (Ct) were used to calculate the fold change.

### Statistical analysis

The data was analyzed and graphs were obtained using GraphPad Prism 5.0 (San Diego, CA, USA). The statistical differences (p<0.05) were measured using one-way analysis of variance (ANOVA) followed by a Bonferroni post-hoc test to compare all means pairwise. The data was represented as mean±SEM.

## Results

### Active ingredients of EthMP extract

The biologically active ingredients from ethanolic extract of MP were estimated through GC-MS analysis. The analysis lead to determination of 17 constituents, listed in Table 2. Results demonstrated that the EthMP extract is dominated with chlorobenzene (entry 1 in Table 2) comprising of 89.23% of total identified contents. Octadecatrieonic acid (1.85%), catechol (1.4%), decamethylcyclopentasiloxane (1.22%), and cyclohexasiloxane (1.15%) constitute remaining bulk of the extract ingredients. The remaining compounds were present with less than 1% of the total ingredients.

**Table 2:** Compounds identified from ethanolic extract of *Mentha piperita*.

RT	Name of Compound	Molecular formula	Molar mass (g/mol)	% area of peak
4.96	Cholorobenzene	C_6_H_5_Cl	112.56	89.23
8.11	Cyclotetrasiloxane	C_23_H_30_O_4_Si_4_	482.8	0.19
11.55	Decamethylcyclopentasiloxane	C_10_H_30_O_5_Si_5_	370.77	1.22
12.46	Catechol	C_6_H_6_O_2_	110.1	1.4
13.49	Carvone	C_10_H_14_O	150.22	0.19
15.07	Cyclohexasiloxane, dodecamethyl	C_12_H_36_O_6_Si_6_	444.92	1.15
15.92	1,4-Pentadiene	C_5_H_8_	68.12	0.48
17.96	5,7-Dimethylenebicyclo [2.2.2]oct-2-ene	C_10_H_16_	136.23	0.69
18.25	3-Isopropoxy-1,1,1,7,7,7-hexamethyl-3,5,5-tris(trimethylsiloxy)tetrasiloxane	C_18_H_52_O_7_Si_7_	577.2	0.50
21.10	2’,4’,4’,6’,6’,8’,8’-Heptamethyltetrasiloxan-2’-yloxy)-2,4,4,6,6,8,8,10,10-nonamethylcyclopentasiloxane	C_16_H_48_O_10_Si_9_	653.3	0.26
25.43	n-Hexadecanoic acid	CH_3_(CH_2_)_14_COOH	256.4	0.93
27.53	Phytol	C_20_H_40_O	296.53	0.27
27.76	9,12-Octadecadienoic acid	C_18_H_32_O_2_	280.44	0.33
27.85	9,12,15-Octadecatrienoic acid	C_18_H_30_O_2_	278.43	1.85
28.11	Allantoic acid	C_4_H_8_N_4_O_4_	176.13	0.25
34.86	1,4-Benzenedicarboxylic acid, bis(2-ethylhexyl) ester	C_24_H_38_O_4_	390.56	0.27
35.36	1,2-Bis(trimethylsilyl)benzene	C_6_H_4_[Si(CH_3_)_3_]_2_	222.48	0.78

RT: Retention Time (Unit: Minutes).

### Motor dysfunctions assessment tests

Baseline behavioral assessment for open field, stepping, stride length, pole-climb down, beam walk and tail suspension tests was conducted to assess normal motor functions of mice.

**Fig 1:**
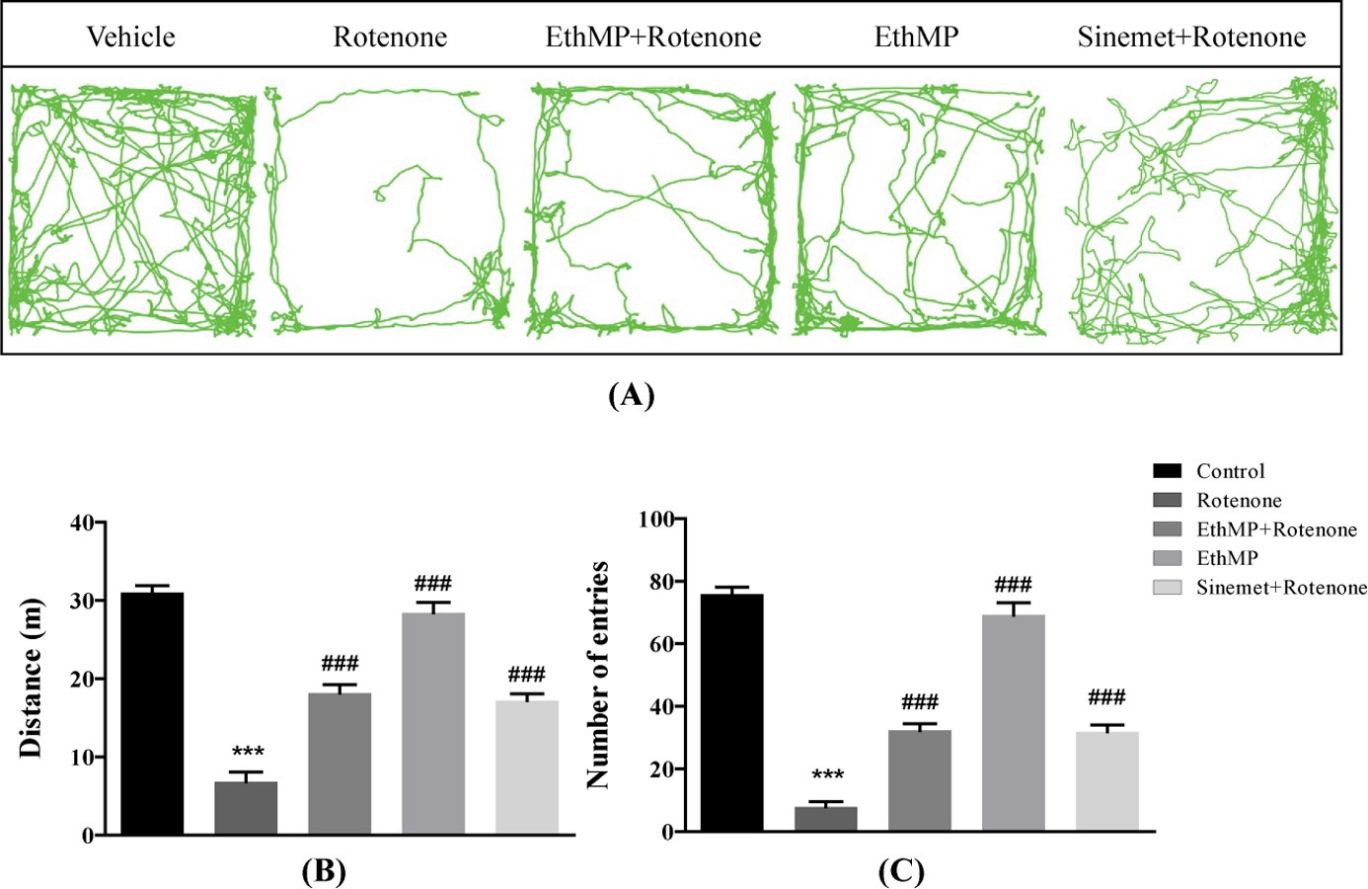
*Mentha piperita* extract prevents the exploratory activities deficits in rotenone-exposed mice. (A) Trajectories of open field demonstrate motor dysfunctions in normal exploratory activities in rotenone-inflicted mice, however a higher extent of locomotion was evident in treatment groups. (B) A significant reduction in total distance travelled in rotenone-treated mice comparing to control, however a significant higher distance covered in treatment groups comparing to rotenone control. (C) A greater reduction in number of entries observed in rotenone-inflicted mice comparing to normal control. A rescue in treatment groups showed potential benefits of the extracts. Data represented as mean ± SEM; n = 9/group, ***p<0.001 vs control, ###p<0.001 vs rotenone control. Oneway ANOVA followed by Bonferroni post-hoc analysis.

Open field test assesses the locomotor activity of mice in an arena. The trajectories of mice in control and treatment groups revealed a sharp decline in motor functions in rotenone treated mice comparing to healthy control, however a maintained locomotor activity was revealed in other groups (Fig 1A). A significant (p<0.001) decline in locomotor activity (6.8±1.2m) was observed in rotenone treated mice comparing to control (30.8±0.9m) and treatment (14.0±1.2m in MP+Rot & 17.4±0.8m in Sin+Rot) groups. While MP+Rot and Sin+Rot administered group significantly (p<0.001) rescued its locomotory activities as compared to rotenone group (Fig 1B). Similarly, a higher number of entries in small squares of open field arena was observed in MP+Rot treated (32±2.1) and Sin+Rot (31±2.04) groups comparing to the rotenone (7±1.6) inflicted mice. However, number of entries in control mice (75±2.1) was comparable with those of extract treated (69±3.8) mice (Fig 1C).

**Fig 2:**
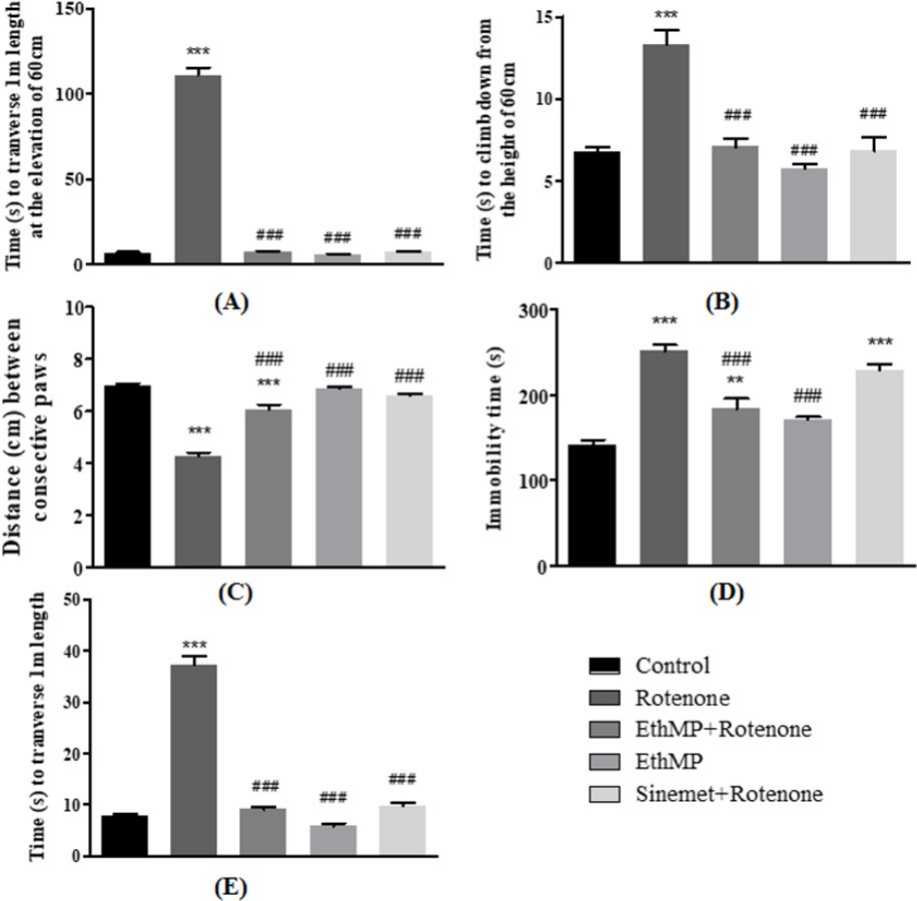
The prevention of motor dysfunctions in EthMP extract treated rotenone-exposed mice. (A) A significant (p<0.001) increase in time to cross a distance of 1m on an elevated beam was observed in rotenone-treated mice, however mice in EthMP and other groups demonstrated significant less time comparing to rotenone-inflicted mice. (B) A significant increase in time taken to climb-down a vertical 50cm distance in rotenone-treated mice demonstrated compromised agility, however significant rescue of mice agility was observed in treated (EthMP & Sinemet) groups. (C) Significant (p<0.001) reduction in stride length of rotenone-treated mice comparing to control mice was rescued in EthMP & Sinement treated groups. (D) The increase in immobility time in rotenone-treated mice was prevented in extract treated mice. (E) Rontenone administration lead to delayed crossing of 1m distance, however, extract treated and Sinemet treated mice covered the same distance earlier. Data represented as mean ± SEM; n = 9/group, ***p<0.001 vs control, ###p<0.001 vs rotenone control. Oneway ANOVA followed by Bonferroni post-hoc analysis.

The beam walk test showed a comparable time taken by control (6.28±0.99s) or MP treated (5.79±0.56s) mice. The time taken to traverse 1 m distance by rotenone group (110.79±4.5s) was significantly (p<0.001) increased as compared to control group (6.28±0.99s). The MP+Rot and Sin+Rot treated mice took significantly (p<0.001) less time (7.12±0.75s and 6.95±0.67s, respectively) to traverse the 1m distance on elevated beam comparing to rotenone group (Fig 2A). A comparable time was taken by control (6.71±0.35s) and MP treated (5.76±0.51s) mice to climb down the vertical pole of 50cm height, however rotenone treated mice descent down in significantly (p<0.001) longer time (13.31±0.86s). A sharp and significant (p<0.001) reduction in time (7.08±0.45s in MP+Rot and 6.83±0.8s in Sin+Rot) in treatment groups to climb-down the pole was observed comparing to rotenone-inflicted mice (Fig 2B).

Stride length measurements in control and MP treated groups were maintained (6.79±0.08cm & 6.85±0.12cm, respectively) and were significantly (p<0.001) reduced in rotenone treatment (4.27±0.15cm) group. Treatment groups showed significantly (p<0.001) higher values for stride lengths (6.05±0.21cm in MP+Rot & 6.57±0.08cm in Sin+Rot groups, respectively) comparing to rotenone treated mice (Fig 2C). Similarly, the extent of immobility while hung from tail was significantly increased (p<0.001) in rotenone administered group (251.48±6.8s), comparing to control (141.04±5.05s). The mice treated with MP+Rot or Sin+Rot demonstrated significantly (p<0.001) lower immobility time (183.67±11.91s and 228.66±6.95s, respectively) from rotenone treated mice (Fig 2D). Control mice crossed a straight corridor of 1m in lesser time (7.81±0.35s) while rotenone inflicted mice crossed the same distance in significantly (p<0.001) longer time (37.3±1.45s), showing compromised skill walking. An improved skill walking (p<0.001) was observed in MP+Rot (9.1±0.4s) and Sin+Rot (9.8±0.5s) groups comparing to rotenone-challenged mice (Fig 2E).

### Enzymatic activity analysis

**Fig 3:**
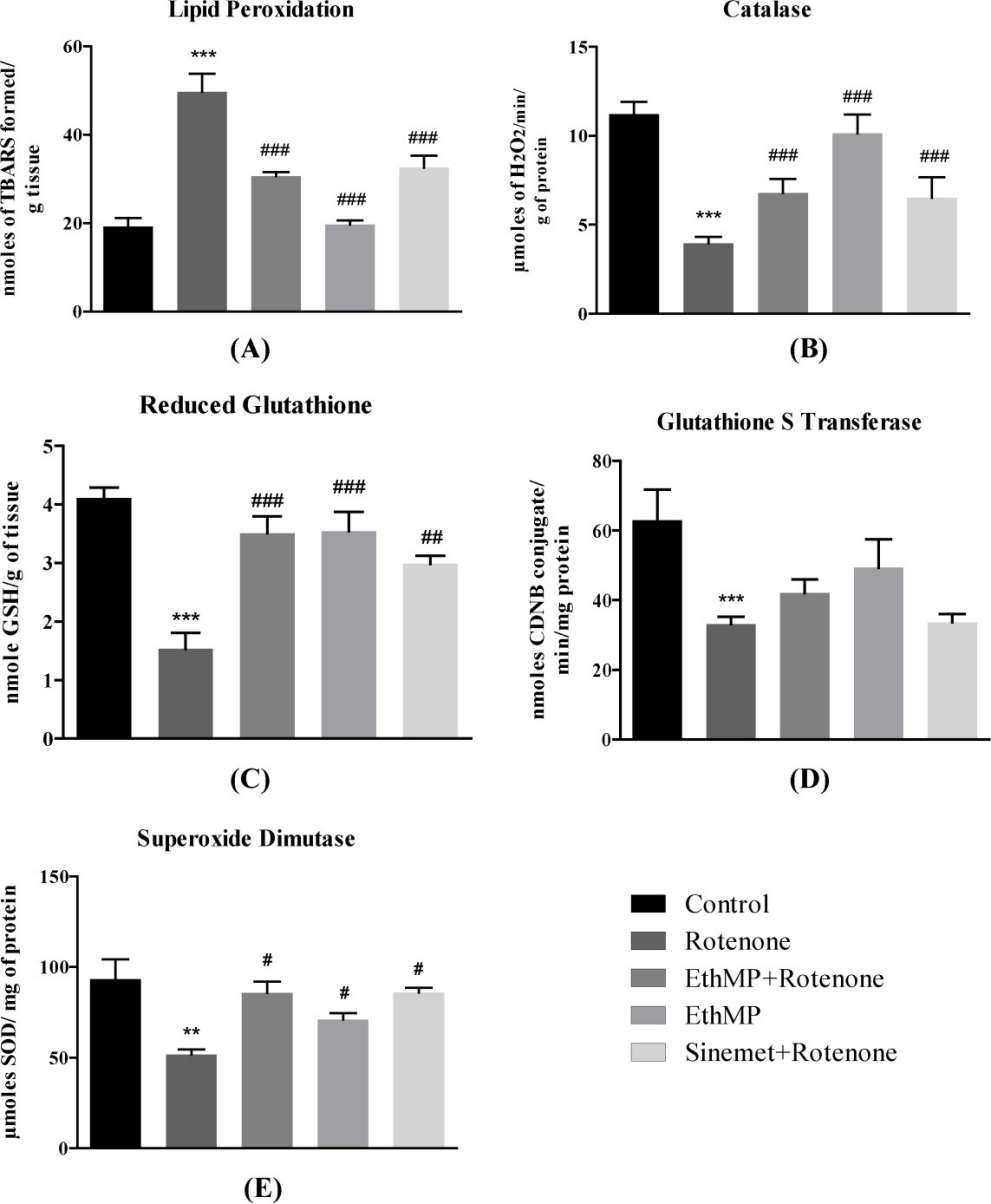
The rotenone-mediated oxidative stress is partially alleviated in EthMP treated mice. (A) Rotenone administration lead to an increased level of LPO in mice brain, however a significant decrease in LPO levels was observed in EthMP & Sinemet groups. (B) A decreased CAT activity was evident in rotenone-inflicted mice and relatively maintained CAT activity in treatment groups comparing to control. (C) Reduced glutathione levels were maintained in treatment groups significantly comparing to the rotenone-inflicted mice. (D) A significant (p<0.001) reduction of GST was evident in rotenone-treated mice. In EthMP extract treated mice & Sinemet treated groups we observed a non-significant increase in GST. (E) A significant reduction in SOD levels in rotenone-treated mice was rescued in extract & Sinemet treated mice. Data represented as mean ± SEM; n = 9/group, **p<0.01, ***p<0.001 vs control,#p<0.05, ##p<0.01, ###p<0.001 vs rotenone control. Oneway ANOVA followed by Bonferroni post-hoc analysis.

Significant (p<0.001) increase in LPO in rotenone treated mice (49.4±3.7μmol/g) comparing to control (19.2±21.8μmol/g) was observed, while the MP+Rot and Sin+Rot treated groups showed marked (p<0.001) decreased in LPO (30.6±0.9μmol/g and 32.5±2.7μmol/g) as compared to rotenone administered group (Fig 3A). The CAT activity was significantly (p<0.001) decreased in rotenone (3.3±0.3nmol/min/g) comparing to control (11.2±0.7nmol/min/g), while it was significantly (p<0.001) increased (6.7±0.8nmol/min/g in MP+Rot & 6.5±1.1nmol/min/g in Sin+Rot) in treatment groups (Fig 3B). GSH level significantly reduced in rotenone group (1.5±0.3nmol/g) as compared to control (4.1±0.17nmol/g) while GSH level was rescued in treatment (3.5±0.14nmol/g in MP+Rot and 2.9±0.13nmol/g in Sin+Rot) groups. MP treated mice demonstrated a non-significant decrease (3.5±0.14nmol/g) in GSH level compared with control (Fig 3C). GST activity was reduced (33.2±1.9nmol/min/mg) in rotenone (p<0.01) comparing to control (62.97±8.7nmol/min/mg), while non-significant (p>0.05) increase was observed in treatment (42.1±3.9nmol/min/mg in MP+Rot and 33.6±2.3nmol/min/mg) groups comparing to rotenone group (Fig 3D). SOD level was significantly (p<0.01) decreased in rotenone group (51.7±2.6μmol/mg) comparing to control (93.3±10μmol/mg). The treatment groups demonstrated significantly (p<0.05) rescued level (85.7±6.2 μmol/mg in MP+Rot and 86±2.5 μmol/mg in Sin+Rot and 70.9±3.6 μmol/mg in MP groups) of SOD (Fig 3E).

### Histopathological analysis

**Fig 4:**
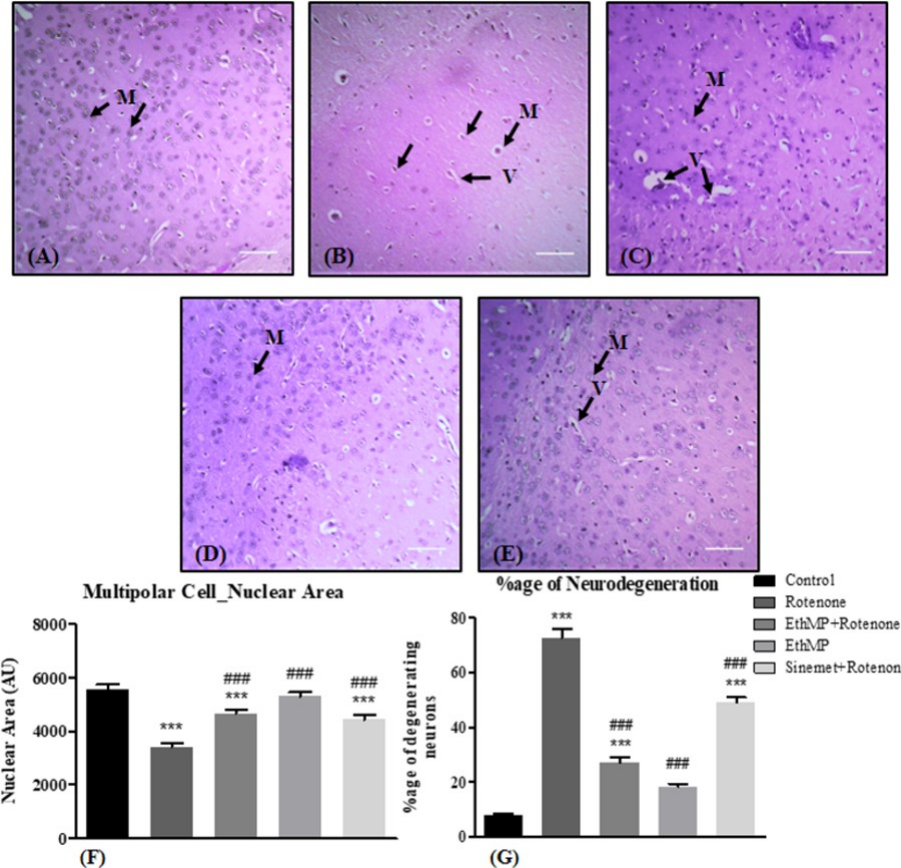
*Mentha piperita* extract imparted neuroprotection in rotenone-treated mice. (A) Hematoxylin & Eosin stained (4μm thick) mid-brain sections viewed at 10x magnification. Scalebar 100μm. (A) Vehicle control mice brain sections revealed a healthy population of mid-brain neurons (black arrows). (B) A considerable population of neurons revealed signs of neurodegeneration (central chromatolysis shown by red arrows). (C) EthMP extract administration to rotenone-inflicted mice showed healthy neuronal morphology. (D) Extract administration imparted no change in neuronal morphology to healthy mice. (E) Sinemet administration reduced the neurodegeneration in rotenone treated mice brain. (F) The nuclear area quantification from the mid-brain sections revealed a significant (p<0.001) reduction in rotenone-treated mice, however a significant rescue of neuronal area was observed in extract & Sinemet treated mice comparing to rotenone administered mice. (G) Quantification of mid-brain cells with signs of neurodegeneration revealed a higher population of such cells in mice exposed with rotenone comparing to treatment groups. Data represented as mean ± SEM; n = 3 sections/group, ***p<0.001 vs control, ##p<0.01, ###p<0.001 vs rotenone control. Oneway ANOVA followed by Bonferroni post-hoc analysis.

Brain sections from control mice showed normal morphology of healthy cell density. A marked reduction (72.56±3.4%) in the number of neurons in the SN region, with neurodegenerative signs of neuronophagia, compromised cell boundaries, shrunken nuclei and central chromatolysis were observed in rotenone-inflicted mice comparing to marginal (7.6±0.82%) neurodegeneration in control mice. The extent of neurodegeneration and neuronal loss was lower in the MP+Rot (48.95±2.08%) and Sin+Rot (27.06±2%) groups (Fig 4A–4E and 4G). The nuclear area was quantified as an assessment of nuclear shrinkage, a marked neurodegenerative sign, and it unveiled a significant (p<0.001) reduction in nuclear area of rotenone-challenged mice mid-brain cells comparing to the control (data represented in arbitrary units). A significant rescue in nuclear area was observed in treatment groups (Fig 4G).

### Dopamine (DA) level in brain tissue

**Fig 5:**
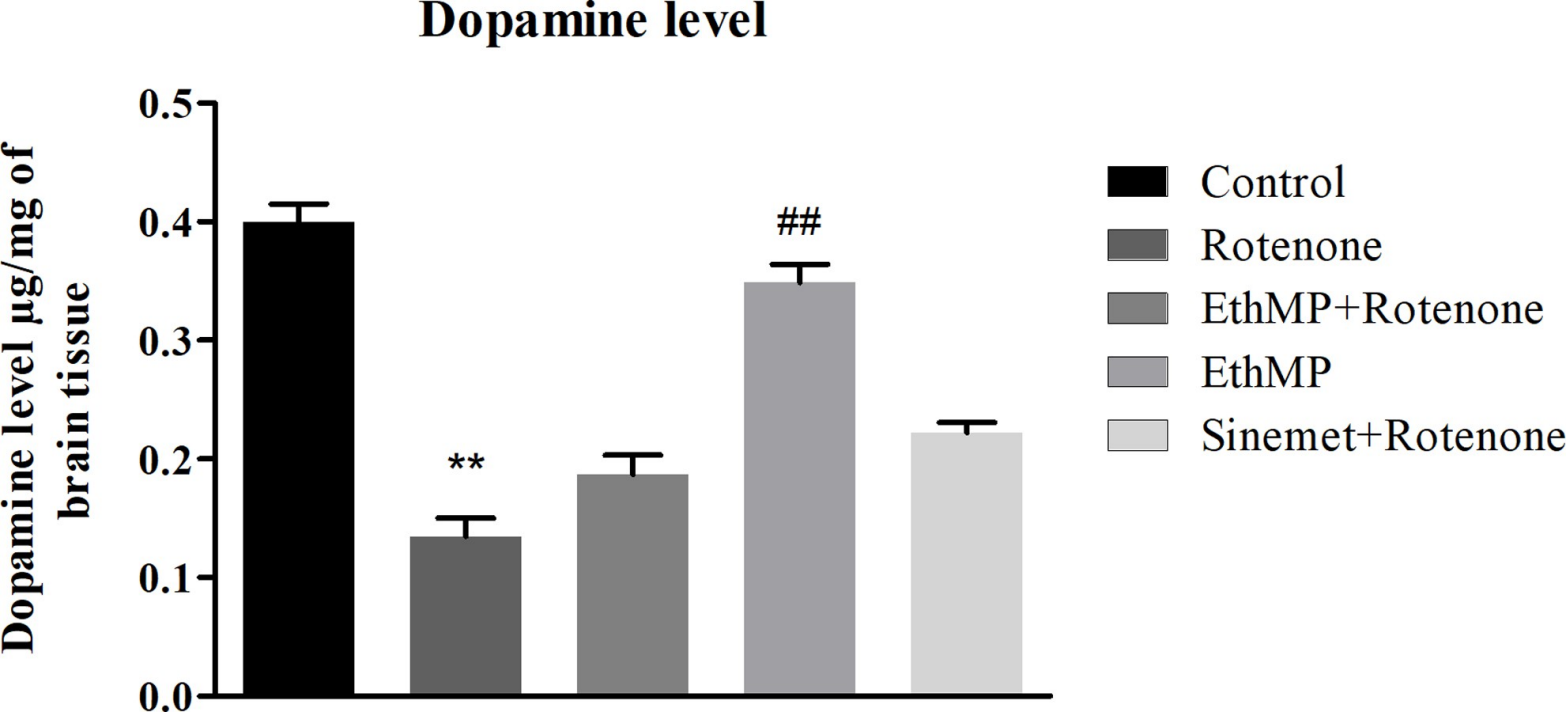
Dopamine levels in mice brain. A sharp decline in dopamine levels in rotenone-challenged mice brain was observed. However, a non-significant increase in dopamine levels was observed in extract & Sinemet treated mice brains exposed with rotenone. The mice treated with EthMP extract only revealed a comparable dopamine levels with vehicle treated mice. Data represented as mean ± SEM; n = 3/group, **p<0.01 vs control, ##p<0.01 vs rotenone control. Oneway ANOVA followed by Bonferroni post-hoc analysis.

DA plays a marked role in motor functions. DA level was markedly decreased (0.13±0.02μg/g) in rotenone administered group comparing to control (0.39±0.03 μg/g) or MP treated (0.35±0.02μg/g) groups. A partial rescue of DA levels in extract treated groups (0.19±0.02μg/g in MP+Rot and 0.22±0.01μg/g in Sin+Rot) reveals a marginal effect of EthMP extract (Fig 5).

### Gene expression analysis

**Fig 6:**
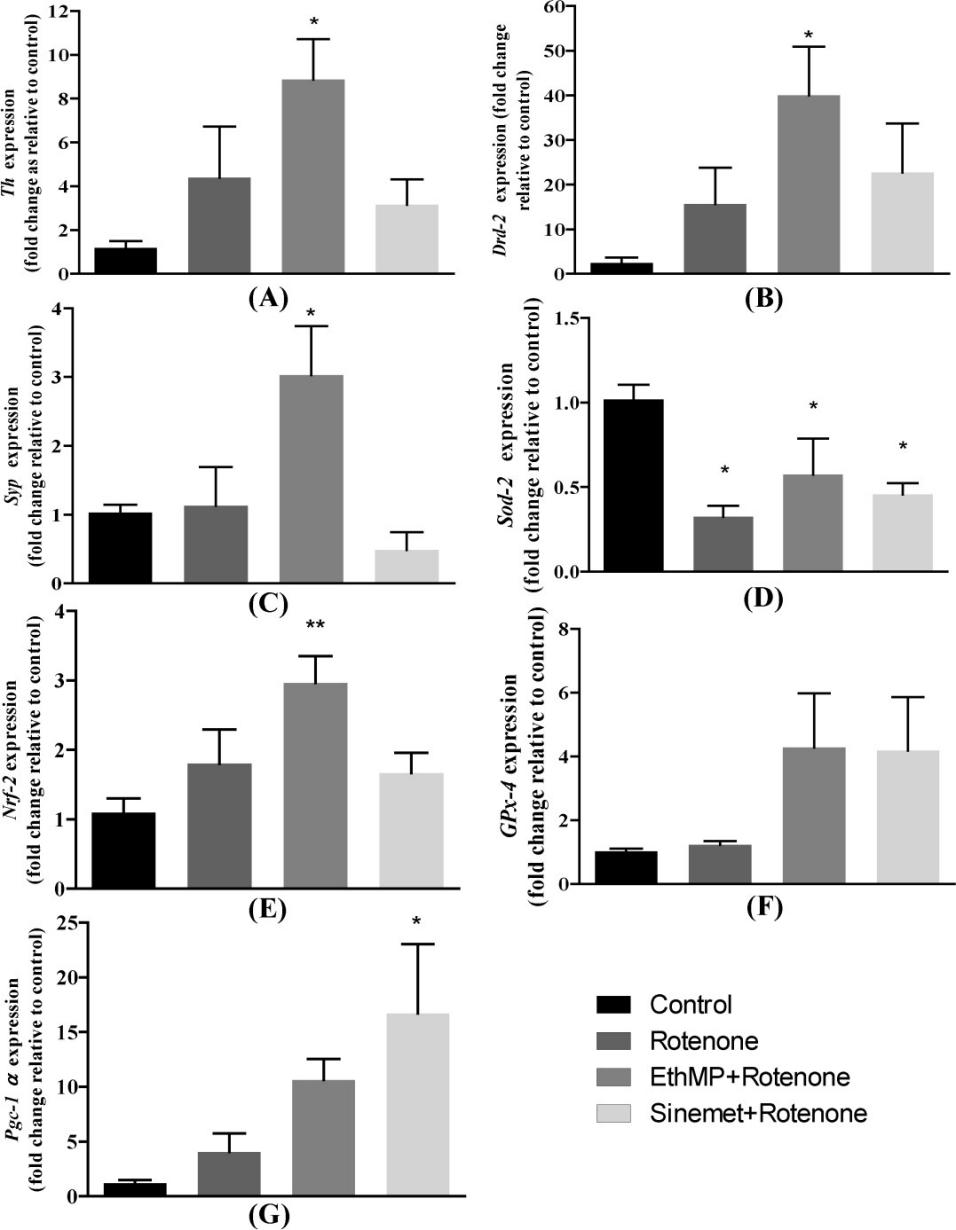
*Mentha piperita* extract modulates the expression of genes involved in dopamine processing, antioxidant capacity, synaptogenesis, and mitochondrial biogenesis. (A) Rotenone-administered mice showed a relatively higher level of Th (tyrosine hydroxylase) expression compared to the control; however, a significant increase (p<0.05) in Th expression was observed in EthMP extract-treated mice and a marginally increased expression in Sinemet-treated mice. (B) Expression of the Drd-2 receptor of dopamine was increased significantly (p<0.05) in EthMP-treated mice; however, marginal upregulation was observed in rotenone- and sinemet-treated groups. (C) Syp expression was comparable in the vehicle control, rotenone-treated, and sinemet-treated mice; however, a significant (p<0.05) increase in the EthMP group was observed. (D) Sod-2 expression was significantly decreased in rotenone-, extract-, and sinemet-treated mice compared to the control. (E) A significant (p<0.01) increase in Nrf-2 expression was observed in the EthMP group compared to the control; however, non-significant increases were observed in the rotenone and Sinemet groups. (F) A comparable expression of GPx-4 was observed in rotenone-treated mice compared to the control, while an increased expression was observed in extract- and Sinemet-treated mice. (G) A modest increase in Pgc-1α expression was observed in the EthMP-treated group; however, a significant increase (p<0.05) was observed in the Sinemet group. Data are presented as mean ± SEM; n = 3 sections/group, *p<0.05, **p<0.01, vs. control. One-way ANOVA followed by Bonferroni post-hoc analysis.

RT-qPCR estimation of Th, Drd-2, Syp, Sod-2, Nrf-2, Gpx-4 & Pgc1-α revealed an altered expression of tested genes in rotenone-challenged mice. Tyrosine hydroxylase (Th) is critical for DA production in brain. Its expression was significantly (p<0.05) increased in the MP+Rot group (7.41±2.63) as compared to control. Rotenone challenged mice and Sin+Rot treated mice demonstrated non-significant (p>0.05) increased expression (4.39±2.33 & 2.05±1.29, respectively) of Th (Fig 6A). Although an increased expression of Th was observed in rotenone-inflicted mice, however HPLC analysis revealed a decreased level of DA in PD mice (Fig 5), suggesting that post-translation events could have hindered the production of DA. Drd-2 regulates synthesis, storage and release of dopamine. In line with Th expression, mice in rotenone treatment group revealed a slightly higher expression of the Drd-2. However the levels were potentiated and significant (p<0.05) in EthMP extract treated mice comparing to other groups (Fig 6B). Synaptophysin (Syp) is involved in regulating hormones via mitochondrial endocytosis. EthMP extract administration significantly increased its expression comparing to control and other groups (Fig 6C).

Sod-2 expression was significantly (p<0.05) decreased in the all treatment groups comparing to control group (Fig 6D), suggesting rotenone perturbs the regulation of Sod-2 in midbrain. Nrf-2 upregulation associates with increased antioxidant capacities. Mice in EthMP extract administration revealed a significant (p<0.01) increased expression comparing to control, suggesting it to promote antioxidant milieu in rotenone challenged condition (Fig 6E). GPx-4 expression remained unaffected in rotenone administration, while a non-significant increase in, EthMP and Sinemet groups was observed (Fig 6G). PGC-1 alpha is considered master regulator of the mitochondria biogenesis and its expression was significantly increased in Sinemet administered mice comparing to control, while a moderate (non-significant) increased expression was observed in rotenone treated mice (Fig 6G).

## Discussion

Repeated exposure of rotenone to rodents induces PD-like motor dysfunctions (bradykinesia, gait alterations and motor coordination deficits) by degeneration of DA producing neurons [37]. It inhibits the mitochondrial complex I of electron transport chain (ETC) and hence elevating oxidative stress leading to PD etiology [38]. Our study harnessed the rotenone-mediated PD motor deficits in mice and observed the therapeutic potential of extract from medicinal herb, *Mentha piperita* through behavioral assays, antioxidant capacities of brain homogenates, extent of neurodegeneration, brain dopamine level determination and expression modulation studies of candidate genes for antioxidant status and synaptogenic activities.

The DA producing neurons of SN play central role in coordinated motor functions [39]. Mounting evidence suggests critical involvement of SN neurons in mediating motor functions with optimal production, release and transmission of DA [40]. However, exposure to environmental toxin may alter the DA circuitry through imparting neurodegeneration and it results in the motor functional deficits [41]. Medicinal plants contain a variety of biologically active ingredients, as previous studies report hexadecanoic acid (Palmitic acid), choloro benzene, catechol and flavonoids from MP extracts and *in vivo* studies reveal their neuroprotective, antioxidant, anti-inflammatory and anti-cancerous properties. In current study, GC-MS based investigation of active ingredients from ethanolic extract of MP revealed 17 ingredients. Thus, our findings were in line with previous investigations [42].

The functional deficits in rotenone-inflicted mice were in consistent with previous studies [43–46]. Current behavioral analyses showed that MP treatment for 21 days improve the locomotor activities in rotenone-challenged mice. MP rescued motor deficits as shown by decreased climb down time and more locomotor activity as evaluated by beam walk, pole, stepping, open field and stride length test. The findings of current study were in line with the previous report [37] suggesting therapeutic potential of EthMP extract in reducing the extent of motor dysfunctions. Furthermore, previous studies employed only open field and rotarod tests to monitor motor functions in animals [47], however in current study additional behavioral tests were used to precisely estimate the disease severity. Behavioral monitoring of the motor functions revealed that MP extract could slow down the rotenone-mediated dysfunctions. Current study suggested a comparable beneficial outcome of EthMP and Sinemet administrations on motor functions in rotenone-inflicted mice.

Elevated ROS generation impairs mitochondrial functions in the SN neurons leading to neurodegeneration [3]. An enhanced LPO in rotenone-inflicted mice brain suggested elevated ROS levels, while prevention of higher LPO in treatment groups suggested the antioxidant capacities of EthMP extract. The decreased levels of reduced glutathione, GST and SOD in rotenone-challenged mice seem to be due to the defective synthesis, utilization and possible neurodegeneration. While mice treated with the EthMP showed significant improvement by lowering level of LPO and increased levels of CAT, GSH, GST and SOD, partially preventing the rotenone-induced oxidative damage. The antioxidant benefits gained in current experiments are in line with the previously reported studies [48, 49] and it supports that MP significantly enhanced the antioxidant defense of body. Thus, it seems that the antioxidant potential of MP leaves extract possibly contribute in rescuing the motor functions in rotenone-inflicted mice.

The mice brain were procured and mid-brain regions were processed for histopathological examination. The mid-brain region of control mice revealed healthy population of neurons with clear cell boundaries, a minor population showed distorted, angulated and shrunken soma consistent with the previous finding [32]. The Hematoxylin & Eosin (H&E) stained brain sections of rotenone-exposed mice showed degenerated eosinophilic body, necrotic and pyknotic changes as reported previously [50]. MP extract significantly ameliorated the neurodegenerative changes triggered by the rotenone. A significantly larger population of mid-brain neurons revealed healthy neuronal morphology [51]. Therefore, it seems MP extract imparted anti-oxidant capacities which enhanced the neuronal survival in mid-brain neurons as reported in similar studies [37, 47].

Improved functional performance, enhanced antioxidant capacities and neuroprotection of mid-brain neurons in MP treated group lead us to quantitative investigation of DA, the crucial neurotransmitter mediating movement functions [52]. Previous study revealed rotenone-mediated mitochondrial complex-1 inhibition lead to the formation of free radicals, and impaired DA levels. DA level significantly decreased in rotenone group comparing to vehicle control, as reported previously [53]. However we observed a modest but non-significant increase in brain DA levels in MP treated mice, suggesting MP plays a little role in increasing DA level.

DA binds with post-synaptic DRD-2 receptor and inhibits adenylate cyclase and downstream signaling [54]. *Drd-2* is expressed at high levels in the principal dopamine projection areas of the CNS. In the current study, the dysregulated expression of dopamine-related genes, *Th* and *Drd-2* in the rotenone-exposed mouse model were indicative of impaired dopamine synthesis, transport, and signaling. However, despite a marginal increase in DA levels in MP-treated mice brain an elevated expression of *Drd-2* suggests the modelling of post-synaptic membrane for augmented DA functions leading to maintained motor functional outcomes under rotenone exposure. Similarly, MP-administered mice revealed an elevated expression of antioxidant marker genes, *Sod-2* and *Nrf-2*, under rotenone exposure. An elevated *Pgc-1α* expression was observed in MP-treated and Sinemet treated mice, pointing to possible augmented biogenesis of mitochondria in mid-brain neurons. Although an increased SOD levels were observed in brain homogenates, we observed no significant increase in *Sod-2* levels in EthMP-treated mice, suggesting an increased efficiency of *Sod-2* rather than its expression.

Collectively, current study reports the therapeutic potential of ethanolic extract of *Mentha piperita* on rotenone-mediated motor dysfunctions in mice. The current study showed improved motor function, antioxidant capacities and reduced neurodegeneration in the SN region, indicating the antioxidant and neuroprotective effects of the MP crude extract. The GC-MS based characterization revealed, many active molecules that may impart neuroprotection, however separate studies are required to unveil their potential.

## Conclusion

The rotenone- mediated motor dysfunctions were significantly prevented in *Mentha piperita* treatment group owing to augmented antioxidant capacities and increased neuronal survival of DA producing neurons in the mid-brain. A partial rescue in DA levels and modulated expression of DA associated genes in MP treatment points to its potential therapeutic capacities that may be harnessed in clinics.

## Supporting information

S1 Raw data (XLSX)
